# Procalcitonin and Risk Prediction for Diagnosing Bacteremia in Hospitalized Patients: A Retrospective, National Observational Study

**DOI:** 10.3390/diagnostics13203174

**Published:** 2023-10-11

**Authors:** Tristan T. Timbrook, Cherilyn D. Garner, Kyle D. Hueth, Gerald A. Capraro, Louise Zimmer, Hari P. Dwivedi

**Affiliations:** 1 BioMérieux, Salt Lake City, UT 84104, USA; cherilyn.garner@biomerieux.com (C.D.G.); kyle.hueth@biomerieux.com (K.D.H.); jerry_capraro14@yahoo.com (G.A.C.); louzimmer84@gmail.com (L.Z.); hari-prakash.dwivedi@biomerieux.com (H.P.D.); 2 Department of Pharmacotherapy, University of Utah College of Pharmacy, Salt Lake City, UT 84112, USA

**Keywords:** procalcitonin, diagnostics, risk factors, bloodstream infection, blood culture

## Abstract

Bacteremia is associated with significant morbidity and mortality. Timely, appropriate therapy may improve clinical outcomes, and therefore, determining which patients benefit from more comprehensive diagnostic strategies (i.e., direct specimen testing) could be of value. We performed an assessment of procalcitonin (PCT) and clinical characteristics in the discrimination of bacteremic hospitalizations. We analyzed 71,105 encounters and 14,846 visits of patients with bacteremia alongside 56,259 without an admission. The area under the receiver—operating characteristic (AUROC) curve for the prediction of bacteremia via procalcitonin was 0.782 (95% CI 0.779–0.787). The prediction modeling of clinical factors with or without PCT resulted in a similar performance to PCT alone. However, the clinically predicted risk of bacteremia stratified by PCT thresholds allowed the targeting of high-incidence bacteremia groups (e.g., ≥50% positivity). The combined use of PCT and clinical characteristics could be useful in diagnostic stewardship by targeting further advanced diagnostic testing in patients with a high predicted probability of bacteremia.

## 1. Introduction

Sepsis results in significant morbidity and mortality and, specifically, is associated with 20% of all global deaths annually [1]. Early appropriate antimicrobial therapy has been established as paramount to improving clinical outcomes in septic patients [2]. Blood cultures are routinely used to direct appropriate therapy in patients with sepsis, but while useful, they are incrementally limited in their clinical impact when considering time to positivity and turnaround time in conventional microbiology processing.

Molecular rapid diagnostic testing (RDT) has been shown to improve the management of patients with sepsis and bloodstream infections (BSIs) [3]. Similarly, the direct specimen RDT has been shown to have the potential to allow for more rapid tailoring of antibiotics in patients with sepsis [4,5]. However, ubiquitous testing among sepsis patients is limited by the laboratory burden in relation to testing costs and staffing resource limitations when performing a related volume of testing. Therefore, targeting testing through diagnostic stewardship to select patients with a high likelihood of BSIs may allow for the application of advanced diagnostics to direct specimens in an effective and efficient manner [6].

Procalcitonin (PCT), a 116-amino-acid precursor to calcitonin, while normally produced by the thyroid in healthy individuals, peaks 12 to 24 h at several-fold higher levels and is produced by a variety of other organs in response to bacterial infection [7]. PCT has been FDA-approved for prognostic use in ICU-admitted sepsis patients and as an aid in antibiotic decision-making in lower respiratory tract infections [8]. Additionally, research on PCT has shown promise in predicting BSIs either alone or in combination with clinical prediction models [9,10]. However, research has been limited due to its single center and/or smaller sample size, thus limiting conclusions on clinical utility. Further research may inform the diagnostic stewardship of direct specimen rapid diagnostics [6].

Our objective was to characterize the performance of PCT when predicting bacterial bloodstream infections across a national inpatient cohort in the US, along with evaluating the additional utility of clinical predictors to identify BSIs, as both approaches could inform diagnostic stewardship efforts toward the effective use of direct specimen RDTs.

## 2. Materials and Methods

### 2.1. Data Source

Hospital encounters were derived from the Premier Healthcare Database (PHD; Premier Inc., Charlotte, NC, USA) [11]. The PHD, which represents 20% of acute care hospitalization nationwide, includes retrospective, observational patient-level encounters with demographics as well as the 10th revision of the International Statistical Classification of Diseases and Related Health Problems (ICD-10), including diagnostic and procedure codes present on admission, invoiced items per day including medications, general laboratory and microbiology data with collection and result alongside the day and times, and facility characteristics. The PHD includes both inpatient and outpatient encounters for over one billion visits over 20 years from currently over 1000 geographically diverse hospitals of varying sizes in both rural and urban areas. This database is compliant with the Health Insurance Portability and Accountability Act. As this study used a fully de-identified database, it was exempt from ethics review under US 45 CFR 46.101(b)4.13 [12].

### 2.2. Study Population

Patients were included if they underwent hospital admission from 1 January 2017 through to 31 December 2019. Adults (≥18 years of age) were selected if they had one or more blood cultures and a PCT level obtained on day one of admission. Encounters were classified by the admission of blood cultures into bacteremic encounters for bacterial etiologies or negative encounters based on culture-based microbiology results. Common contaminants (e.g., coagulase-negative *Staphylococcus* spp., *Micrococcus* spp., etc.) were excluded due to the absence of data on vitals (e.g., heart rate and temperature) provided by the PHD, and therefore, we were unable to adjudicate likely contamination by standardized definitions [13,14]. Fungal and mycobacterial bloodstream infections were also excluded due to the discrimination performance of PCT being specific and robust for typical bacterial infections [15,16].

### 2.3. Outcomes and Definitions

Descriptive data were analyzed for the cohort, including baseline patient and hospital characteristics, in addition to patient encounter characteristics. Chronic disease comorbidities were evaluated using the Charlson Comorbidity Index, which was derived from standardized ICD-10 codes [17]. Similarly, admission diagnoses were evaluated using ICD-10 codes (Supplementary Table S1). Laboratory values were selected based on their Logical Observation Identifiers Names and Codes (LOINC^®^; Supplementary Table S2) and evaluated based on clinically relevant abnormalities. Maximum values of lactate and serum creatinine on admission were assessed, while minimum values of albumin and platelets were evaluated. WBCs were assessed for the presence of leukopenia (<4000 cells/mm^3^) or leukocytosis (>12,000 cells/mm^3^). Inpatient mortality was determined by hospital encounter discharge records.

The discrimination performance of PCT for diagnosing bacteremia was assessed for the overall cohort. Subgroups were analyzed among those admitted with a diagnosis (overall cohort, pneumonia, pyelonephritis, and febrile neutropenia) and were evaluated based on relevance from previous studies [9,10]. Likewise, age, comorbidities, admission diagnosis, and laboratory-based clinical predictors of bacteremia on admission were examined through the derivation of a regression model based on previously described prediction models [9,10]. The predictors evaluated included an admission or present on admission diagnosis (pyelonephritis, pneumonia, febrile neutropenia), PCT level, elevated serum creatinine (>2 mg/dL), elevated lactate (>2 mmol/L), leukocytosis or leukopenia, low platelets (<150,000/mm^3^), and low albumin (≤3 g/dL) [9,10]. The erythrocyte sedimentation rate (ESR), C-reactive protein (CRP), and lactate were not included, as they are not routinely used in practice in the majority of facilities for sepsis patients (i.e., missing data for at least 40% of the study population) and were thus prohibitive to imputation. All time-varying predictors (i.e., laboratory values) were assessed based on day one of admission labs.

### 2.4. Statistical Analysis

Patient, hospital, and encounter-related characteristics were summarized using the median (IQR) and number (percentage). The performance of PCT to predict bacteremia was assessed using sensitivity, specificity, and the area under the receiver operating characteristic curve (AUROC). An AUC of 0.5 reflects the discrimination capacity consistent with chance, while 1 reflects perfect discrimination. The optimized cutoff for PCT was determined using Youden’s index value.

Additional encounter predictors were evaluated along with PCT in an unconditional logistic regression model for the evaluation of bacteremia [9]. Multivariable logistic regression was performed with the least absolute shrinkage and selection operator (LASSO), a penalized least squares method, with candidate variables for model development and model selection based on a lambda shrinkage parameter with best performance in a ten-fold cross-validation [18]. Final variables were modeled with logistic regression, as LASSO could bias final model coefficients toward zero based on its soft thresholding property [18,19,20]. The sample size was assessed using the pmsampsize package for prediction modeling using 14 candidate parameters with a potential prevalence of 5% bacteremia and an average derivation model of 0.79 c-statistic from previous models [21]. The estimated required samples and events were at least 2125 and 107, respectively. Single imputation was performed if data were missing. Model performance was evaluated for discrimination (C-statistic) and calibration (agreement between predicted and observed using calibration slopes).

We performed all analyses in R, version 4.0.1 (R Foundation for Statistical Computing, Vienna, Austria). Comorbid conditions were mapped from ICD-10s using the *comorbidity* package (v0.5.3), ROC curve analysis with the *pROC* package (v1.18.0), and LASSO regression with the *glmnet* package (v4.1-4) [17,22,23]. Prediction model derivation was reported according to the transparent reporting of a multivariable prediction model for the individual prognosis or diagnosis (TRIPOD) checklist [24].

## 3. Results

We identified 71,105 patients meeting the eligibility criteria of at least one procalcitonin level and blood culture obtained on the day of admission; 14,846 were bacteremic, and 56,259 were non-bacteremic encounters. Patient and hospital characteristics among bacteremic and non-bacteremic encounters were overall similar (Table 1). The median age of the cohort was 68 years (IQR 56–79), and 49.8% were female. The Charlson Comorbidity Index was the same across groups (median 3, IQR 2–4); however, bacteremic patients had a higher proportion of diagnoses for chronic renal disease (33.2% vs. 28.6%) and chronic hepatic disease (11.4% vs. 7.1%) compared to non-bacteremic patients. The majority of patient encounters occurred in hospitals with ≤500 beds (36.1%) and in urban settings (92.3%).

The clinical characteristics of patient encounters can be found in Table 2. The majority of encounters occurred in hospitalist or internal medicine wards as the admission service (78.0%). Among admission or present-on-admission diagnoses, as described in previous derivation or validation models for predicting bacteremia, pneumonia, and sepsis were common, occurring in 42.1% and 42.7% of encounters, respectively. Notably, bacteremia occurred less commonly among pneumonic patients (34.8% vs. 44.0%) and was more common among sepsis patients (80.3% vs. 32.8%). Abnormal laboratory values were more common among bacteremic patients, and specifically, the median PCT was 2.44 (IQR 0.45–13.81) among bacteremic patients and 0.19 (0.07–0.73) among non-bacteremic patients. Finally, the length of stay (4 vs. 6 days) and mortality (6.0% vs. 11.0%) were lower among non-bacteremic patients than bacteremic patients.

**Figure 1:**
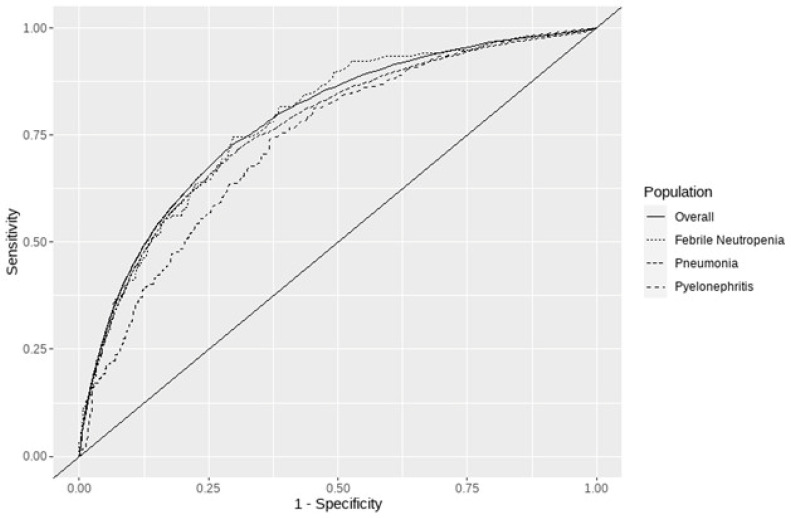
AUROC curves overall and for patient admission diagnosis.

The diagnostic performance of PCT for bacteremia had good discrimination with an AUROC of 78.2% (95% CI, 77.9 to 78.7). Discrimination performance decreased among pneumonia and pyelonephritis admission or present-on-admission diagnoses (Figure 1). Sensitivity-balanced (72.9%) and specificity-balanced (70.2%) discrimination performance was maximized at a cutoff threshold of 0.53 ug/mL. Additionally, a regression model was developed (Table 3) based on clinical characteristics and lab results including PCT, which reflected moderate discrimination performance (C-statistic 80.7%) and calibration (Supplementary Figure S1). In the absence of PCT in this model, discrimination performance was slightly lower (C-statistic 79.4%), and calibration was similar. Prediction model regression equations can be found in the Supplementary Materials (Table S3).

Bacteremia incidence varied when evaluated using PCT-only stratified cutoffs, a prediction model with PCT stratifications, and a prediction model without PCT (Table 4). For PCT only, levels of >2 to <10 ng/dL and >10 ng/dL were associated with a bacteremia incidence of 40% or more compared to 20.9% for the overall cohort. Similarly, using the clinical prediction model without PCT, a medium (25–75%) and high (>75%) risk of bacteremia predicted the probability of bacteremia incidence in patients as 42% or more. When using both PCT and the clinical prediction model with stratified cutoffs together, a bacteremia incidence of 53% or more was noted with medium risk and PCT > 2 ng/dL, while all high-risk patients had an incidence of 70% or higher.

## 4. Discussion

In our comprehensive cohort study of 71,105 hospitalized patients from whom we obtained blood cultures and procalcitonin, we demonstrated a moderate diagnostic discrimination performance when predicting bacteremia for both procalcitonin testing and clinical prediction modeling. Our findings showcase a moderate level of performance for both methodologies. When considering the immense challenge and critical importance of timely bacteremia detection, the potential utility of either approach in guiding advanced diagnostic testing from blood cultures becomes evident. While either approach may be beneficial in driving advanced diagnostic testing from blood cultures, the use of both approaches at stratified ranges can allow increased incidence risk groups who are most likely to benefit from advanced diagnostic testing to be targeted. These evaluations reflect a diagnostic stewardship approach of enriching patient selection for advanced diagnostic testing to ensure the right test for the right patient at the right time [25]. Furthermore, we observed that PCT discrimination performance across admission diagnoses was mostly comparable, with the exception of pyelonephritis and pneumonia, which had lower performance. This insight underscores the complexities and variables inherent in bacteremia diagnostics. Additionally, we found high proportions (60.3%) of bacteremic patients with a PCT > 10 ng/dL, reinforcing the value of PCT as a diagnostic marker.

Previous studies of clinical prediction modeling when predicting bacteremia have resulted in an AUROC of 0.60–0.83, while pooled estimates of PCT have reflected an AUROC of 0.79 [9,10]. We observed discrimination performance to be similar among both PCT and clinical prediction models. In a systematic review and meta-analysis of procalcitonin for diagnostic accuracy on bacteremia for 58 studies and 16,514 patients (3420 with bloodstream infection), cut-off values for PCT varied between 0.10 ng/dL and 17 ng/dL (median 0.5 ng/dL) [9]. Similarly, in our overall cohort, we observed 0.53 ng/dL as the threshold for balancing sensitivity and specificity. This balance assumes that false positives and false negatives are equally important, which might not be applicable to all clinical scenarios [26]. For instance, a febrile neutropenia patient admitted to an ICU on pressors could benefit from a low threshold that increases sensitivity. By contrast, a young adult floor patient without comorbidities likely has a threshold favoring increased specificity. Additionally, when used to evaluate advanced diagnostic testing decisions, this threshold may be a function of prevalence for disease or a potential for therapy modification based on advanced diagnostic. Moreover, continuous prediction models without specific thresholds could help facilitate individual-level decision-making in certain use cases, allowing for personalized medicine [26]. As we look ahead, there is a compelling need for research that dives deep into crafting prediction models tailored to specific advanced diagnostics and patient populations, ensuring that the chosen thresholds resonate with the unique demands of each clinical context.

Recent research has evaluated the unique characteristics of patients with lower PCT levels (<2, 0.5 to 2, and <0.5 ng/dL) and bacteremia [27], noting that these patients are difficult to identify based on observed attributes. In the context of a clinical presentation that is consistent with potential bacteremia, these PCT levels could make appropriate workup and diagnosis challenging. We found that bacteremia incidence was 9.1% and 25.4% for <0.5 and 0.5 to <2 ng/dL, respectively, but higher likelihoods could be targeted through clinical prediction modeling to >24% and >40% for these cutoffs if restricted to medium- and high-risk-prediction-model patients, reflecting the synergistic value of PCT and clinical prediction models for determining patients who are likely bacteremic. This combined approach not only augments diagnostic accuracy but also underscores the importance of a multi-faceted strategy when managing complex clinical scenarios.

The realm of direct specimen testing often enters intricate territories. Direct specimen testing can be problematic, as testing is driven by often subjective judgments of who benefits from testing or their perceived pre-test probability of a bloodstream infection [4,6]. If optimal testing is not approached through diagnostic stewardship, a lower diagnostic yield and avoidable increased costs without meaningful clinical impacts may result. To recognize these inherent challenges and address this issue, we performed a retrospective evaluation of the diagnostic accuracy of PCT and a validation of clinical prediction for bacteremia. Our findings illuminate a promising path: harnessing the insights from these tools offers a strategic direction for advanced testing. This aligns with previous research in this area, which has found PCT to be an avenue for improving direct specimen testing with SeptiFast real-time PCR (Roche Diagnostics GmbH, Mannheim, Germany) in a more cost-effective manner [6]. While we evaluated a retrospective national cohort using conventional culture diagnostics, we believe our data build on this literature and aforementioned systematic reviews through our combination approach of PCT and clinical prediction models, which allows a variety of bacteremia incidence groups to be targeted with increased precision. As the implementation of advanced diagnostics can occur across varied, heterogenous settings with competing needs, limited resources, and clinical practice, the implementation and prioritization of advanced diagnostics should be tailored. Therefore, our suggested approach can allow flexibility based on these needs. In an envisioned diagnostic framework, patients exhibiting a predicted incidence of bacteremia greater than 50% could be selectively prioritized for advanced direct specimen testing. This strategic direction could harness the synergistic power of both PCT and clinical risk prediction models, as illustrated in a hypothetical diagnostic pathway in Supplementary Table S4. To truly realize the potential of these integrated approaches, it is imperative that future research engages in subsequent external validations of the prediction model. Furthermore, future investigations focusing on these approaches with the practical clinical utility of specific advanced diagnostic methodologies could be instrumental in refining and substantiating our proposed strategies.

Our study has several limitations. As our data were retrospective, the blood cultures, procalcitonin, and other labs were not systematically collected and thus subject to clinical variability and bias. However, the performance reported herein likely reflects pragmatic real-world utility. While our study is derived from 1200 diverse hospitals and is likely nationally representative, generalization to specific hospitals should be undertaken with caution and proceed with local validations. In contrast with our present study, most previous studies are limited to single centers in a specific geographic setting with samples of 200–2000, and, therefore, our data likely reflect a typically expected discrimination performance. In our research, we did not incorporate data related to erythrocyte sedimentation rate (ESR) or the C-reactive protein (CRP) as predictive markers for bacteremia. The rationale behind this exclusion stemmed from several considerations. First, these particular biomarkers, while valuable in assessing systemic inflammation, have only sporadically been incorporated into bacteremia prediction models in prior studies [10]. This limited inclusion might reflect their broader role in indicating inflammation rather than specifically identifying bacterial infections. Second, existing research using extensive national US cohorts underscores a notable trend: approximately 80% of clinical encounters do not involve the use of these biomarkers for this purpose, suggesting a prevailing clinical practice pattern that often omits ESR and CRP in the bacteremia diagnostic process in the US [28]. While our study sought to mirror current practices and prevailing research trends, we acknowledge the potential utility of these markers. Future investigations might benefit from a comprehensive evaluation of ESR and CRP, potentially revealing additional insights into their diagnostic relevance in bacteremia. We also did not analyze pediatric patients. We focused on adults as labeling-supported and clear cutoffs that exist to guide evaluations and analysis [8]. By contrast, pediatric patients, particularly neonates and young infants, may have elevated PCT in the absence of bacterial infection [29]. While we believe our approach of either clinical prediction, PCT, or a combination thereof offers merit in predicting bacteremia and driving advanced diagnostic testing in pediatrics as well, specific models and thresholds should be evaluated in future research for this population. Finally, our goal was to find reliable predictors to drive advanced diagnostic testing, and the current study only provides a potential approach toward that goal. Future research should evaluate the use of PCT, clinical prediction models, or a combination thereof as a diagnostic stewardship and pre-analytical strategy to drive advanced diagnostic testing.

## 5. Conclusions

In our study, spanning 71,105 hospitalized patients, the pivotal role of procalcitonin (PCT) and clinical prediction modeling in bacteremia diagnosis is underscored. Both individually and in tandem, these methods offer significant diagnostic accuracy. Elevated PCT levels in a notable 60.3% of bacteremic patients highlight its diagnostic potential. Furthermore, the combined strength of PCT and clinical models points to a nuanced, tailored approach for patient selection in advanced diagnostic testing—epitomizing diagnostic stewardship. While direct specimen testing brings its own set of challenges, our research underscores the necessity for a more integrated diagnostic approach. As the landscape of bacteremia diagnostics evolves, our findings set the stage for future research, emphasizing the importance of refining and validating these tools in diverse clinical contexts.

## Figures and Tables

**Table 1:** Patient and hospital characteristics.

Characteristic	All Patients (*N* = 71,105)	Bacteremic (*N* = 14,846)	Non-Bacteremic (*N* = 56,259)
Age	68 (56–79)	67 (56–78)	68 (56–79)
Sex			
Female	35,392 (49.8)	6937 (46.7)	28,455 (50.6)
Male	35,713 (50.2)	7909 (53.3)	27,804 (49.4)
Race/ethnicity			
Black	10,014 (14.1)	1954 (13.2)	8060 (14.3)
White	53,436 (75.2)	11,020 (74.2)	42,416 (75.4)
Other	7655 (10.7)	1872 (12.6)	5783 (10.3)
Comorbidities			
Charlson Comorbidity Index	3 (2–4)	3 (2–4)	3 (2–4)
Chronic renal disease	21,000 (29.5%)	4936 (33.2%)	16,064 (28.6%)
Chronic hepatic disease	5704 (8.0%)	1698 (11.4%)	4006 (7.1%)
Chronic obstructive pulmonary disease	29,406 (41.1%)	4602 (31.0%)	24,804 (44.1%)
Congestive heart failure	24,446 (34.4%)	4921 (33.1%)	19,525 (34.7%)
Malignancy	9057 (12.7%)	1962 (13.2%)	7095 (12.6%)
Hospital Beds			
0–99	5685 (8.0)	1080 (7.3)	4605 (8.2)
100–199	11,242 (15.8)	2374 (16.0)	8868 (15.8)
200–299	8660 (12.2)	1812 (12.2)	6848 (12.2)
300–399	12,022 (16.9)	2708 (18.2)	9314 (16.6)
400–499	7853 (11.0)	1550 (10.4)	6303 (11.2)
>500	25,643 (36.1)	5322 (35.8)	20,321 (36.1)
Hospital location			
Rural	5497 (7.7)	1305 (8.8)	4192 (7.5)
Urban	65,608 (92.3)	13,541 (91.2)	52,067 (92.5)

Data as median (IQR) or no. (%).

**Table 2:** Clinical characteristics of patient encounters.

	All Patients (*N* = 71,105)	Bacteremic (*N* = 14,846)	Non-Bacteremic (*N* = 56,259)
Admitting service			
Family practice	5148 (7.2)	1118 (7.5)	4030 (7.2)
Hospitalist/ Internal medicine	55,461 (78.0)	11,331 (76.3)	44,130 (78.4)
Pulmonary/ Critical care	3034 (4.3)	877 (5.9)	2157 (3.8)
Other	4877 (6.9)	1063 (7.2)	3814 (6.8)
Unspecified	2585 (3.6)	457 (3.1)	2128 (3.8)
Admitting diagnosis			
Febrile neutropenia	513 (0.7%)	147 (1.0%)	366 (0.7%)
Pneumonia	29,937 (42.1%)	5162 (34.8%)	24,775 (44.0%)
Pyelonephritis	960 (1.4%)	559 (3.8%)	401 (0.7%)
Sepsis	30,359 (42.7%)	11,924 (80.3%)	18,453 (32.8%)
Laboratory values			
PCT, ng/dL	0.27 (0.08–1.55)	2.44 (0.45–13.81)	0.19 (0.07–0.73)
Leukopenia or leukocytosis	33,543 (47.2%)	8478 (57.1%)	25,065 (44.6%)
Platelets < 150,000/mm^3^	12,530 (17.6%)	4124 (27.8%)	8406 (14.9%)
Lactate > 2 mmol/L	17,488 (24.6%)	5287 (35.6%)	12,201 (21.6%)
Albumin ≤ 3 g/dL	22,141 (31.1%)	6384 (43.0%)	15,757 (28.0%)
Creatinine > 2 mg/dL	13,749 (19.3%)	4186 (28.2%)	9563 (17.0%)
Clinical outcomes			
LOS	5 (3–8)	6 (4–10)	4 (3–7)
Mortality	4987 (7.0)	1639 (11.0)	3348 (6.0)

Data as median (IQR) or no. (%). Abbreviations: ICU, intensive care unit; PCT, procalcitonin; LOS, length of stay; WBC abnormality as <4000 cells/mm^3^ or >12,000 cells/mm^3^; ICU admit on day of admission. Among all patients, the absence of lab results occurred for WBC 12.7%, platelets 14.4%, lactate 44.1%, albumin 17.5%.

**Table 3:** Predictive model for bacteremia.

Predictor Variable	Crude OR (95% CI)	Adjusted OR (95% CI)	*p*-Value *
Baseline characteristics			
Age	0.9976 (0.9965–0.9987)	1.0 (1.0013, 1.0027)	0.047
Diabetes	1.17 (1.13, 1.22)	1.09 (1.04, 1.14)	<0.001
Hepatic disease	1.68 (1.59, 1.79)	1.26 (1.17, 1.35)	<0.001
Malignancy	1.06 (1, 1.11)	0.92 (0.86, 0.98)	<0.001
COPD	0.57 (0.55, 0.59)	0.77 (0.73, 0.8)	<0.001
Renal disease	1.25 (1.2, 1.3)	1.04 (0.99, 1.09)	0.155
Congestive heart failure	0.93 (0.9, 0.97)	1.09 (1.04, 1.14)	<0.001
Admission diagnosis			
Sepsis	8.37 (8.01, 8.75)	7.06 (6.74, 7.4)	<0.001
Pneumonia	0.68 (0.65, 0.7)	0.55 (0.52, 0.57)	<0.001
Pyelonephritis	5.45 (4.79, 6.2)	2.73 (2.36, 3.15)	<0.001
Febrile neutropenia	1.53 (1.26, 1.85)	1.17 (0.93, 1.46)	0.18
Laboratory			
Creatinine >2 mg/dL	1.92 (1.84, 2)	1.06 (1, 1.12)	0.056
Albumin ≤ 3 g/dL	1.94 (1.87, 2.01)	1.3 (1.24, 1.36)	<0.001
Platelets < 150,000/mm^3^	2.19 (2.1, 2.29)	1.68 (1.6, 1.77)	<0.001
Leukocytosis or leukopenia	1.66 (1.6, 1.72)	1.11 (1.07, 1.16)	<0.001
Procalcitonin, ng/dL	1.03 (1.03, 1.03)	1.02 (1.02, 1.02)	<0.001

* Variable selection by LASSO depends on performance in the model for a ten-fold cross-validation; therefore, all variables in the final logistic model may not be significant.

**Table 4:** Bacteremia incidence via PCT only and clinical prediction models.

		Predicted Risk without PCT			Predicted Risk with PCT		
PCT Level (ng/dL)	PCT only *N* = 71,105	Low (<25%) *N* = 44,767	Med (25–75%) *N* = 26,182	High (>75%) *N* = 156	Low (<25%) *N* = 46,137	Med (25–75%) *N* = 23,813	High (>75%) *N* = 1155
<0.5	9.1% (3935/42,810)	-	-	-	5.6% (1938/34,828)	24.1% (1993/8278)	100% (4/4)
0.5< to <2	25.4% (3059/12,060)	-	-	-	13.2% (893/6783)	41.0% (2159/5267)	70% (7/10)
>2 to <10	40.4% (3557/8811)	-	-	-	21.4% (740/3460)	52.5% (2800/5331)	85% (17/20)
>10	60.3% (4295/7124)	-	-	-	27.1% (289/1066)	63.0% (1828/4937)	80.0% (897/1121)
Overall	20.9% 14,861/71,105	8.2% (3693/44,767)	42.1% (11,029/26,182)	79.5% (124/156)	8.4% (3860/46,137)	42.3% (10,061/23,813)	80.0% (925/1155)

## Data Availability

PINC AI™ Healthcare Data (PHD) is commercially licensed and only available from Premier under license.

## Supplementary Materials

The following supporting information can be downloaded at https://www.mdpi.com/article/10.3390/diagnostics13203174/s1. Figure S1: Calibration of bacteremia model with clinical characteristics and lab results excluding PCT; Figure S2. Calibration of a bacteremia model with clinical characteristics and lab results including PCT; Table S1: Admitting or present on admission diagnoses from ICD-10 codes; Table S2. Logical Observation Identifiers, Names and Codes (LOINC) laboratory codes; Table S3. Prediction Models. Table S4. Decision making on advanced diagnostic testing using direct specimens.
