# Supplementary material for: Procalcitonin and Risk Prediction for Diagnosing Bacteremia in Hospitalized Patients: A Retrospective, National Observational Study
Source: Diagnostics (Basel). 2023 Oct 11;13(20):3174. doi: 10.3390/diagnostics13203174 (PMC10605738; doi:10.3390/diagnostics13203174)
Supplement: Supplementary file 1 [file diagnostics-13-03174-s001.zip › diagnostics-2651923-supplementary.pdf]

Supplemental Materials

Table S1. Admitting or present on admission diagnoses from ICD-10 Codes

Variable	Codes
Pneumonia	481, 481, 482.2, 482.30, 482.31, 482.32, 482.39, 482.84, 482.9, 483.0, 483.1, 483.8, 485, 486, J13, J18.0, J18.1, J18.8, J18.9, J15.0, J15.3, J15.4, J15.5, J15.6, J15.7, J15.8, J15.9, J16.0, J16.8, J14, 480, 480.1, 480.2, 480.3, 480.8, 480.9, 482.4, 482.41, 482.42, 482.49, 482.81, 482.82, 482.83, 482.89, 483, 484.3, 484.5, 484.6, 484.7, 484.8, 487, 488.01, 488.11, 488.81, J10.00, J10.01, J10.08, J11.0, J11.00, J11.08, J12.0, J12.1, J12.2, J12.3, J12.8, J12.81, J12.89, J15.1, J15.20, J15.21, J15.211, J15.212, J15.29, J69.0
Fever	R50, R50.2, R50.8, R50.81, R50.82, R50.83, R50.9
Neutropenia	D70, D70.0-4, D70.8-9
Sepsis	R65.20, R65.21, 670.20, 670.22, 670.24, 995.91, 995.92, A02.1, A22.7, A26.7, A32.7, A40, A40.0, A40.1, A40.3, A40.8, A40.9, A41-2, A54.86, B37.7, O03.37, O85, O86.04, P36
Pyelonephritis	590.0, 590.1, 590.10, 590.8, 590.80, 590.81, A02.25, D86.84, N10, N11.1

Table S2. Logical Observation Identifiers, Names and Codes (LOINC) Laboratory Codes

Platelets	26515-7, 777-3, 778-1, 5907-1, 74464-9, 79427-1, 97995-5
Procalcitonin	75241-0, 33959-8, LG15749-1
WBC	58410-2, 26464-8, 6690-2, 804-551383-8, 12227-5, 33256-9, 26464-8, 30406-3, 57021-8, 57022-6, 57782-5,

Creatinine	2160-0, 44784-7, 35203-9, 14682-9, 21232-4, 38483-4, 59826-8, 77140-2, 24362-6, 50261-7, 24320-4, 24321-2, 24322-0, 24323-8, 45064-3, 45065-0, 51990-0, 70219-1, 88843-8, 89044-2
Albumin	1751-7, 61151-7, 61152-5, 54347-0, 62235-7, 62234-0, 76631-1, 77148-5 24322-0, 24323-8, 45065-0, 89044-2
Lactate	2524-7, 32693-4, 51829-0, 30242-2, 2519-7, 30241-4, 14118-4, 19239-3, 19240-1, 2518-9, 2519-7, 32132-3, 32133-1, 35245-0, 59032-3,

Table S3. Prediction Models

PCT+predictors model	-2.693935592 (Intercept) + 0.001342232*AGE + 0.016094408*PCT + 0.083899681*diabetes + 0.230911834*liver_disease - 0.088039088*malignancy -0.265200533* COPD + 0.03776216*renal_disease + 0.082137661*CHF + 1.003462724*Admit_pyelo + 0.154997402*Admit_FN + 1.954755840*Admit_sepsis + 0.054753876*Abnormal_scr + 0.260894165*Abnormal_alb + 0.520389720*Abnormal_plt + 0.108123071*Abnormal_WBC
Predictors only model	-2.6465545788 (Intercept) + 0.0005363365*AGE + 0.0615012849*diabetes + 0.1856434769*liver_disease - 0.0929392763*malignancy - 0.2810399153*COPD + 0.0123805899*renal_disease + 0.0708894530*CHF + 1.0333367679*Admit_pyelo + 0.1375249469*Admit_FN + 2.0675191862*Admit_sepsis + 0.2489875526*Abnormal_scr + 0.3187883713*Abnormal_alb + 0.5844923683*Abnormal_plt + 0.1158177503*Abnormal_WBC

Figure S1. Calibration of bacteremia model with clinical characteristics and lab results excluding PCT

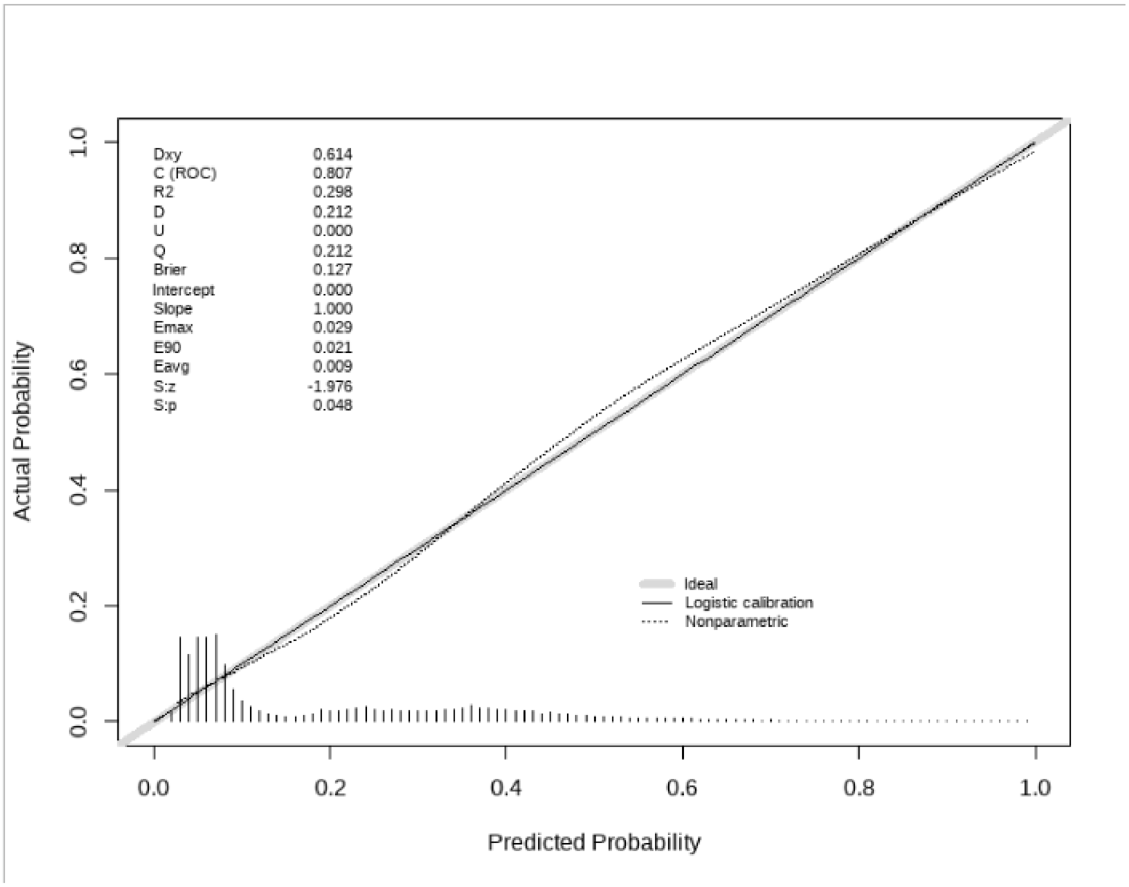

Figure S2. Calibration of bacteremia model with clinical characteristics and lab results including PCT

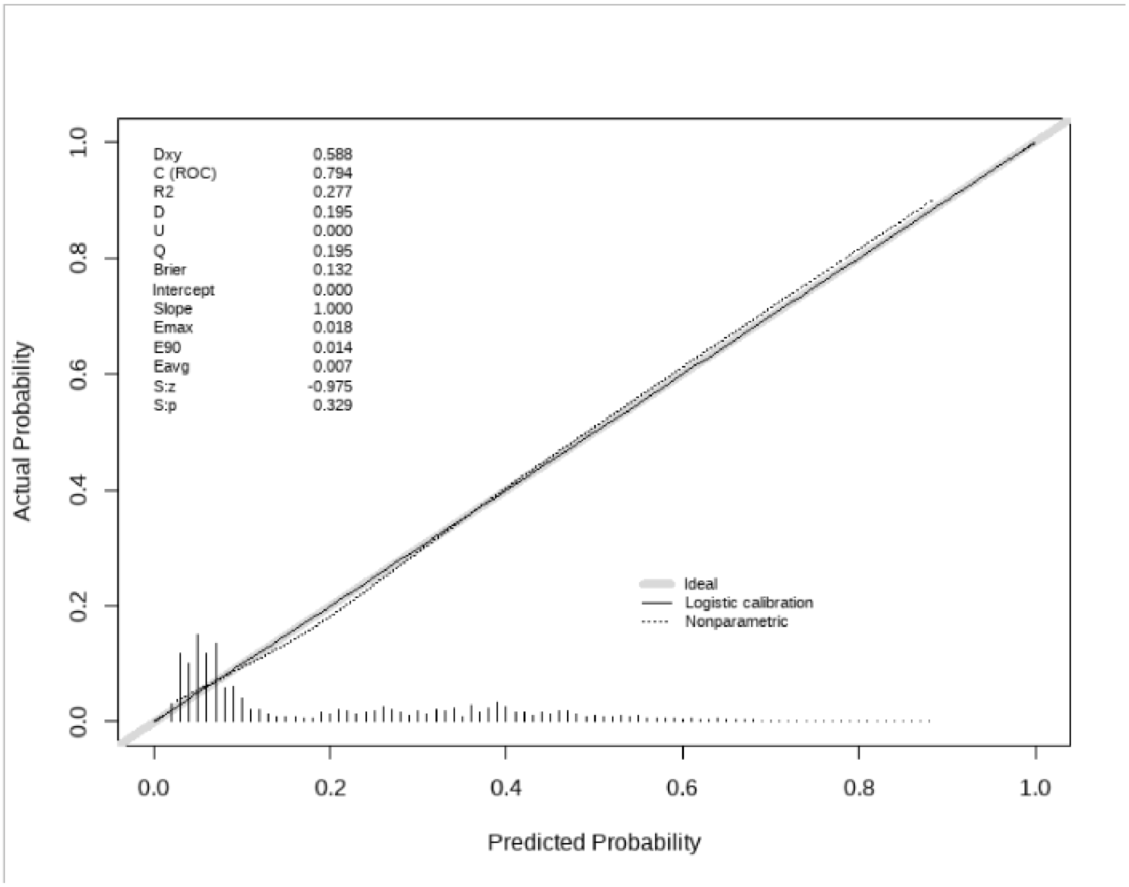

Table S4. Decision making on advanced diagnostic testing using direct specimens

		Predicted Risk without PCT			Predicted Risk with PCT		
PCT Level (ng/dL)	PCT only	Low (<25%)	Med (25-75%)	High (>75%)	Low (<25%)	Med (25-75%)	High (>75%)
<0.5	No	-	-	-	No	No	Yes
0.5< to <2	No	-	-	-	No	No	Yes
>2 to <10	No	-	-	-	No	Yes	Yes
>10	Yes	-	-	-	No	Yes	Yes
Overall	-	No	No	Yes	-	-	-
